# Development and Pilot Testing of a Food Literacy Curriculum for High School-Aged Adolescents

**DOI:** 10.3390/nu13051532

**Published:** 2021-05-01

**Authors:** Lyndsey D. Ruiz, Marcela D. Radtke, Rachel E. Scherr

**Affiliations:** 1 Department of Nutrition, University of California, Davis, Davis, CA 95616, USA; ldruiz@ucdavis.edu (L.D.R.); mdradtke@ucdavis.edu (M.D.R.); 2 Center for Nutrition in Schools, University of California, Davis, Davis, CA 95616, USA

**Keywords:** food literacy, adolescent, curriculum development, experiential learning, nutrition education, healthy lifestyles

## Abstract

Adolescent obesity and poor diet quality are increasingly prevalent and could be mitigated with attainment of food literacy. However, as these programs for adolescents are lacking, the purpose of this project was to develop a food literacy curriculum for high school-aged adolescents. The curriculum was designed in accordance with food literacy attributes and components utilizing Backward Design, Social Cognitive Theory, and Constructivism. After expert committee review, pilot testing was completed in two low-income communities by a trained facilitator and observer. Detailed observations were collected during pilot testing to assess achievement of learning objectives. Modifications were made to lesson procedures as required. The resulting curriculum, *Teens CAN: Comprehensive Food Literacy in Cooking, Agriculture, and Nutrition*, contains 12 modules of experiential lessons and application activities within three topics. Agriculture lessons concentrate on the food supply chain and food environments; nutrition lessons include food groups while focusing on nutrients of concern for underconsumption; and cooking lessons include food safety, budgeting, and preparation. *Teens CAN* provides a comprehensive and necessary approach to advancing food literacy in adolescents. Future directions include assessing dietary implications after participating in *Teens CAN* lessons and employment of an innovative two-tiered cross-age teaching model.

## 1. Introduction

Obesity is a multifactorial disease that is challenging to treat, requiring several considerations and components encouraging behavioral modifications [1]. Adolescent obesity can have lasting consequences on health as it is associated with adulthood obesity and thus may affect long-term quality of life and lead to development of chronic diseases [1,2,3,4,5,6,7]. Prevalence of adolescent obesity has progressively increased over the last several years and adolescents consistently have the highest rates of obesity among youth [8]. In 2016, 1 in 5 adolescents were classified as obese, with prevalence of obesity highest in Hispanic and non-Hispanic Black adolescents with 25.9% and 25%, respectively [8,9]. These values were above the 20.6% average for adolescents aged 12–19 years and higher than that of non-Hispanic White youth within the same age group [9]. Providing guidance for healthy lifestyle choices may limit risk factors perpetuated by childhood obesity. Youth who reduce incidence of obesity mitigate the associated increased risk of adulthood chronic disease, instead exhibiting comparable risk to that of youth who were never obese [4,5]. This supports an urgent need to educate adolescents as they transition to experiencing more autonomy in food choices and other lifestyle behaviors that arise with emerging adulthood [10].

Poor diet quality may contribute to the high prevalence of adolescent obesity [3]. Over 50% of youth had poor diet quality in 2016 [11]. Youth are well below meeting dietary recommendations despite having quite high nutritional requirements to support a period of immense growth [12,13,14]. Diet quality progressively decreases as youth advance in age, with high school-aged adolescents having lower diet quality compared to youth of elementary school age [11,15]. In particular, adolescents aged 14–18 years do not meet recommendations for consumption of fruits, vegetables, and whole grains [11,13,16]. Adolescents in the lowest quartiles of intake for each food group tend to continue having low levels of intake into adulthood [16]. Consistently and of particular concern, youth from low-income communities tend to have the poorest diet quality [11,15].

While not the only consideration, poor diet quality of adolescents may be attenuated with advancement of food literacy [17]. Beyond the focus of traditional nutrition education, food literacy requires understanding of food procurement and preparation [18,19]. Food literacy involves having the knowledge and skills necessary to make healthy dietary choices and comprises 11 components within 4 domains [18] and 15 attributes within 5 categories [19]. Many nutrition education programs utilize some of these elements, however few incorporate all. Components of food literacy were extrapolated from surveying experts and young adults [18] and attributes were identified through a scoping review of the literature [19]. Food literacy components [18] are specific while attributes are more thematic [19]. For example, the component “determine what is in a food product, where it came from, how to store it and use it” [18] encompasses several attributes related to food selection and preparation [19]. These elements include both critical knowledge, such as understanding nutrition-related information, and functional knowledge, wherein application of knowledge through skills and choices is essential, that intersect to aid in developing and maintaining healthy food behaviors [18,19,20]. Education in one domain or category is not sufficient for achieving food literacy, instead scaffolding of knowledge and skills from the various topic areas is required [19]. A systematic and narrative review of food literacy programs for high school-aged adolescents found that interventions at least 4 weeks in length that included opportunities for advancement in knowledge and self-efficacy were most likely to affect short-term dietary behavior [21]. Additionally, several recommendations for implementing food literacy interventions have been identified [22]. Such recommendations include utilizing settings where adolescents normally congregate and engaging in weekly experiential activities that provide opportunities for application of food-related knowledge and skills [22]. Furthermore, it is recommended to tailor the program approach to the specific age group being targeted and to provide opportunities that support positive youth development [22]. Despite the need, especially considering the high rates of obesity, food literacy programs targeting older adolescents are limited [22,23]. This dearth in food literacy programming prevents adolescents from gaining knowledge and skills needed to make healthy food choices as young adults and perpetuates unhealthy food practices observed during childhood [10].

Previous findings from a study conducted within the 4-H Youth Development Program (4-H) found that adolescents did not have foundational knowledge to effectively lead garden-enhanced nutrition and cooking lessons [24]. Focus groups completed in Australia found that adolescents had some prior food-related knowledge from participation in year-long required courses, but had limited opportunities to apply that knowledge through food preparation [25]. Participants in the focus groups expressed an interest in increasing food literacy through home economics courses [25]. Home economics courses are increasingly rare in the United States and topics relevant to food literacy are often categorized into health courses. However, national Health Education Content Standards [26] include a plethora of topics that must be covered in one semester (the length of a typical health class in the United States) and thus completing food literacy education outside of the typical school day may be more feasible. Informal settings, such as afterschool programs, encourage the acquisition of knowledge through lifelong, life-wide, and life-deep learning, which incorporate the people, places, and culture that every individual brings to a learning environment, whether in or outside of a formal classroom [27]. This is especially helpful for learning concepts that directly impact learners’ everyday lives and require synthesis of various prior experiences in conjunction with newly acquired information. Unlike traditional classroom learning, which mostly applies to meeting objectives of school, such as completing exams and assignments, informal learning objectives can be directly applicable to knowledge needed for daily life activities [28,29]. With this, the objective of this project was to develop a comprehensive food literacy curriculum for high school-aged adolescents to be implemented through afterschool programs.

## 2. Methods

### 2.1. Curriculum Development

Developing curricula based on theories and recognizing needs of the target population are recommended for maximum efficacy [30,31]. Furthermore, curricula that focus on behavior change and skill development in addition to knowledge attainment tend to be more successful [31,32,33]. Therefore, Social Cognitive Theory [34] and Constructivism [35] were selected as theoretical frameworks while also considering the Social Ecological Model [36]. Social Cognitive Theory is widely utilized in nutrition interventions [31] and conceptualizes dietary change with consideration for the intersection of personal, environmental, and behavioral factors [34]. Constructivism functions through a community of learners engaged in active discourse, allowing for creating knowledge together with the goal of deep and sustained learning [35]. The Social Ecological Model [36] provided context for factors that affect food choices of adolescents at various levels including local access, peer influence, and preparation skills, among others. The food literacy curriculum was developed following systematic procedures previously utilized to design a garden-enhanced nutrition curriculum for a multicomponent school-based nutrition intervention called the Shaping Healthy Choices Program [37,38]. The process began with assembling a development team including three experts in the overarching topic areas, agriculture, nutrition, and cooking, which were deemed necessary for development of food literacy through consolidation of the components [18] and attributes [19], and 13 undergraduate interns. The experts collectively had extensive knowledge in curriculum development, nutrition, sustainable agriculture, food systems, garden-based education, recipe development, and cooking techniques.

To develop the curriculum with intention, Backward Design [39] was employed. The first step of Backward Design is to *identify desired results* [39], which was implemented through determining concepts that youth should learn after participating in the curriculum lessons [39]. Interns were instructed to independently search for learning concepts by reviewing reputable resources, including peer-reviewed literature, government reports, and educational standards. Under supervision of the relevant content expert, learning concepts were grouped and consolidated into the three topic areas in addition to being reviewed for alignment with aspects of food literacy. This was proceeded by the second step of Backward Design, *determine acceptable evidence* [39], which was employed to develop learning objectives guided by authentic assessment [40]. Authentic assessment accompanies Constructivism [35], requiring a product or performance and encouraging collaboration while developing new knowledge that can be applied to other tasks [40]. The learning objectives were written in accordance with higher levels of Bloom’s Taxonomy [41] to promote retention of knowledge and skills gained from participation in the lessons.

The final step of Backward Design is to *plan learning experience and instruction* [39]. Primary lessons were designed in accordance with the 5-Step Experiential Learning Cycle [42] and utilizing guided inquiry [43]. Experiential learning [44] was selected as the pedagogical approach to foster active learning through experience and development of skills within each lesson. Furthermore, lesson objectives were aligned so that knowledge and skills acquired during each lesson could be applied to one another and built upon as lessons progressed. Experiential learning [44] complements constructs of Social Cognitive Theory [34] by drawing from previous experiences and encouraging learning from others participating in the experience while also building behavioral capacity and self-efficacy through achieving learning objectives. Each intern developed an experience to achieve each learning objective and facilitated their lesson with the larger group for initial feedback. Immediately following the lesson, all interns completed guided reflection documents [37,45] to facilitate constructive discussion of aspects that worked well and ones that needed improvement. Lessons were revised and implemented a second time following the same method with the full curriculum development team. Application components were also drafted to allow for learning opportunities within an agricultural space, such as a school garden, and for hands-on cooking opportunities. Additionally, home application lessons were created to extend content beyond the experience while also supporting growing and preparing food at home.

### 2.2. Pilot Testing

Prior to pilot testing with youth, the curriculum was reviewed by an expert committee. This committee included individuals with expertise in curriculum development and learner-centered pedagogy in addition to experts in the three topic areas. Nine members participated in the committee and were contacted based on known content knowledge and recommendations; most committee members were university and Cooperative Extension academics. Minor edits were made following the expert committee review to improve clarity and background information provided as a facilitator introduction to each lesson. No modifications were made to the lesson procedures at this time.

Pilot testing was conducted with high school-aged adolescents during afterschool hours in two low-income communities in Northern California. It was important to pilot test with representatives of the intended target audience to ensure that authentic assessments were challenging but achievable [40,43]. The first pilot took place in a suburban community at a community center in collaboration with two afterschool programs. Lessons were conducted at the community center and nearby community garden three days per week, over five weeks, to an average of 12 participants. The second pilot occurred at a high school during afterschool hours in a rural community. This pilot was delivered in the multipurpose room and school garden once a week, for 12 weeks, to an average of eight participants. Participation in both pilots was completely voluntary and concluded after all lessons were delivered. Participants of both pilots received home kits for hydroponically growing lettuce and basic cooking supplies. The curriculum lessons and application activities for both pilots were facilitated by an educator trained in learner-centered pedagogy who was not involved in the initial curriculum development. The principle author of the curriculum was also present at each pilot lesson to serve as an observer. Both the facilitator and observer completed observation sheets that were modified from the previous method [45] to include additional structure in accordance with each component of the 5-Step Experiential Learning Cycle [42]. Observations encompassed elements that worked well in helping participants achieve the predetermined authentic assessments for each lesson and areas requiring improvement. Additionally, comments regarding level of engagement, such as number of youth on-task and the proportion of youth actively completing lesson assignments, were included in the *procedure* and *sharing*, *processing*, and *generalizing* segments. The observation sheet also featured an open notes section where ideas for improvement could be documented. The facilitator and observer met the following day after each lesson to compare observation sheets and come to a consensus on suggested lesson revisions. Following each completed pilot, informal group interviews were held with participants to gain qualitative insight into acceptability and enjoyment of the lessons. Revisions were made as needed and implemented at the subsequent pilot. Data were not collected from participants given that the objective of the pilot tests was to assess whether learning objectives were achieved for each lesson. Procedures for the pilot tests were approved as exempt by the University of California, Davis Institutional Review Board.

## 3. Results

The resulting curriculum is entitled *Teens CAN: Comprehensive Food Literacy in Cooking, Agriculture, and Nutrition* (*Teens CAN*). *Teens CAN* was developed to align with the components [18] and attributes [19] of food literacy in an effort to be as comprehensive as possible (Table 1). *Teens CAN* meets several California educational standards, in particular Next Generation Science Standards in regard to life and earth sciences [46], Common Core State Standards for speaking and listening [47], and many nutrition and physical activity Health Education Content Standards [26].

*Teens CAN* started with 13 lessons, however, the first pilot test observations suggested combining two of the nutrition lessons for succinctness. While all learning concepts and objectives (Table 2) were retained, three lesson procedures were modified as the original procedures did not allow for achieving the identified authentic assessments and thus participants were unable to meet the learning objectives. Additionally, youth indicated feeling less engaged during these lessons compared to others. Insufficient time was initially dedicated to developing application activities, resulting in almost all being revised to better suit each lesson concept. Following the second pilot, only additional minor edits to improve clarity throughout the curriculum were required. All participants of the second pilot were able to achieve the learning objectives through acceptable evidence of learning as detailed in Table 2. Further, observations indicated that youth were adequately engaged during the lessons. Results from the informal group interview suggested that youth enjoyed the learner-centered approach of the lessons. The final curriculum contains 12 modules, four within each topic area, that feature experiential and application lessons. The agriculture application activities allow for working within an agricultural space, whether a community or school garden, or another designated space for growing food. Each module can be completed within two hours and includes detailed background information and facilitation tips. While training in learner-centered pedagogy is recommended, and frequently provided for Cooperative Extension educators, these features allow for implementation with minimal experience. To accompany the curriculum, a guide for developing and maintaining an agricultural space was written and integrated into the introduction.

Agriculture lessons were designed to feature the food system, including different agricultural systems, inputs, and innovations that have contributed to establishing current practices. Aspects of urban agriculture were also incorporated, which comprises smaller-form farming in addition to community and school gardening. Agriculture applications for these lessons entail touring a local farm, or having a local producer visit the agricultural space to share information about their production and exploring inhabitants found in the agricultural space to investigate their impact on the growing environment. Another application involves working in the agricultural space with and without modern day equipment in order to understand how innovations within agriculture have shaped modern procedures. Home application activities include interviewing individuals with roles in the food supply chain, learning more about insects and animals that are involved in agriculture, growing produce at home, and mapping out one’s own neighborhood to assess food availability and access.

Nutrition lessons begin with practice categorizing foods into food groups, as defined by MyPlate [48], and then move to identifying macronutrients and micronutrients and assessing overlaps with the food groups. The other nutrition lessons include learning how to meal plan with the nutrients of concern for underconsumption in adolescents [49] and analyzing nutrition messages in media. Agriculture application activities for nutrition lessons involve planning a snack using items grown in the agricultural space, testing soil quality, establishing a compost pile, and making sustainable pesticides using household products. Home application activities include meal planning utilizing recommendations for MyPlate [48] food group consumption and the nutrients of concern for underconsumption [49]. Additionally, home application activities entail assessing Nutrition Facts Labels on products found at home and analyzing a nutrition-related advertisement.

The first cooking lesson focuses on food safety, including proper handwashing and setting up a safe work space. Each subsequent lesson and culinary application activity begin with a reminder to wash hands that is followed with verification of practicing food safety throughout the experience. Other cooking lessons entail advancing knife skills and practice utilizing basic cooking techniques and equipment. Meal planning in accordance to shopping in season and within budgetary constraints is featured in cooking lessons as well. Recipes provided within the curriculum are vegetarian to limit food safety concerns regarding temperature and to introduce adolescents to plant-based protein sources. Use of produce grown in the agricultural space for the recipes is encouraged. Furthermore, all recipes were intentionally developed with low-cost ingredients, including canned and frozen products, and regularly available food items as to not limit low-income communities from preparing the dishes. In addition to encouraging youth to make recipes, adapting as needed to meet family and cultural preferences, cooking home applications activities entail examining and avoiding potential food safety hazards at home. Additionally, home applications included meal planning utilizing seasonal produce and scaling recipes to feed their families.

## 4. Discussion

*Teens CAN* aims to improve food literacy, and consequently diet quality, of high school-aged adolescents, with the ultimate intent to reduce and prevent obesity. Being food literate requires the skills and knowledge necessary to grow, buy, and cook food while considering health, so that empowered individuals can make the healthier choice when given the option [18,19]. However, food literacy is a relatively emergent concept with accompanying limitations. While improvements in dietary outcomes have been observed in adolescent interventions aimed at improving attributes of food literacy [17], long-term implications for diet quality and obesity prevalence are lacking [21]. Additionally, food literacy is a complex construct with multiple interrelated factors, making assessment of food literacy challenging [19,21,50]. As such, a comprehensive evaluation tool is yet to be developed for this age group [19,50]. Nevertheless, the potential of food literacy is worth exploring given that youth aware of food growing practices and regionality of produce are more likely to consume fruits and vegetables [51]. Additionally, studies have shown that youth are more likely to consume healthier diets when they are involved in the food preparation process [52,53]. This was observed during *Teens CAN* pilot testing as participants regularly harvested and sampled produce growing in their agricultural space. Furthermore, youth who were apprehensive to taste new foods at the beginning were open to trying included recipes by the end.

The approach of *Teens CAN* includes three topic areas of agriculture, nutrition, and cooking with experiential and application lessons aimed at improving knowledge and skills related to healthy eating. *Teens CAN* is inexpensive to implement, requiring mostly printed materials provided within the curriculum and common school supplies. Additionally, recipes provided within the cooking lessons feature low-cost produce available year-round as well as shelf-stable items. The curriculum employs activities and concepts that require critical thinking from participants, which adolescents are capable of completing [31]. In addition to application activities that incorporate an agricultural space, each module includes home application activities to nurture further learning. These activities additionally provide opportunity for appropriate adaptions, such as those for cultural considerations, to make adoption of new practices more viable.

*Teens CAN* was designed and tested following a similar approach to *Discovering Healthy Choices* [54] for the Shaping Healthy Choices Program, which has similar theming and includes some food literacy components [18,37,38]. The Shaping Healthy Choices Program has shown improvements in nutrition knowledge and weight status among other healthy behaviors [55,56,57]. Similar agriculture concepts to that of *Teens CAN*, such as components of the food system and food security, were included in another curriculum, *Sprouting Healthy Kids*, designed for middle school students in Texas, that was found to improve participant fruit and vegetable intake [58].

As *Teens CAN* is aimed at improving food literacy of high school-aged adolescents, findings from focus groups where adolescents ranked aspects of food literacy according to importance strongly influenced lesson concepts [25]. Adolescents ranked food and nutrition knowledge among the most important aspects of food literacy for them to develop healthy eating patterns [25]. Focus groups identified that adolescents did not pay attention to food labels or dietary guidance due to not understanding their application [25]. With this, use of Nutrition Facts Labels and recognizing nutrients of concern, as identified within the Dietary Guidelines for Americans [49], were focused on in the *Teens CAN* nutrition lessons. The Dietary Guidelines for Americans recommends consuming healthy eating patterns with adequate intake of essential nutrients through a varied diet that incorporates each food group [49]. Though the curriculum was written to align with the 2015–2020 Dietary Guidelines for Americans [49], recommendations for adolescents did not substantially change with the newest edition [59]. Food cards within nutrition lessons included foods typically considered unhealthy and high in empty calories since adolescents frequently consume these foods [13], as well as healthy alternatives to allow for comparison. Additionally, whole fruits, vegetables, and grains were heavily featured to encourage consumption as adolescents are well below meeting recommendations for these foods [11,13,16]. With adolescence being a time of increased autonomy [14], all primary nutrition lessons and application activities were written to support adolescents planning for meeting their own nutritional needs. Furthermore, to tailor lessons to adolescents, all characters presented in lesson activities were high school-aged adolescents.

Culinary skills education has been called to be incorporated into nutrition education for application of concepts through hands-on food preparation [60]. While adolescents from the previously mentioned focus groups ranked food preparation skills as of low importance [25], other findings suggest that limited opportunities for hands-on food skills practice are a hindrance leading to low food literacy as young adults [10]. Due to this, primary learning concepts involved enhancement of food skills and opportunities to prepare food. Adolescents also acknowledged that while budgeting and shopping for food were not immediately important in their current life stage, these concepts would be later in life [25]. With this, budgeting and shopping for food was added as one of the cooking lesson concepts.

Cooking programs provide an enjoyable experience that introduces youth to preparing and tasting dishes containing new, frequently healthier, foods [61,62]. Culinary application activities in *Teens CAN* feature cultural cuisines, advise consumption of produce grown in the agricultural space, and allow opportunities for participants to have independence in ingredient selection. Participation in cooking programs also motivates youth to continue practicing learned food skills at home [61], which has been associated with more nutritious eating patterns [60]. Adolescents who participate in food preparation at home are more likely to continue enjoying cooking and preparing healthier dishes as emerging adults [63].

The application activities integrated in *Teens CAN* may indirectly improve adolescent health as community gardening experience was found to be positively associated with willingness to try fruits and vegetables in low-income high school students from an urban community [64]. Additionally, participating in farm to school related activities has been associated with willingness to try fruits and vegetables in addition to improving nutrition knowledge and self-efficacy [65]. Providing opportunities for involvement in agriculture, even if just through gardening, is important given that childhood, in combination with recent, gardening for first-year college students was found to be associated with higher fruit and vegetable consumption compared to those who have never gardened [66]. *Teens CAN* lessons introduce adolescents to agriculture concepts and encourages growing food at home through application activities. This could perhaps establish a mechanism for adolescents to continue gardening into later adolescence and adulthood.

*Teens CAN* has since been translated into Spanish. Having the curriculum available in Spanish helps reach more participants as almost 40% of the population in California is of Hispanic descent [67]. This allows for the curriculum to be utilized for development of language education, such as district-level programs that encourage multilingualism. Additionally, the materials allow for adolescents to engage Spanish-speaking family members with the home application materials.

Planned implementation of *Teens CAN* was designed to align with recommendations for older adolescent food literacy programs [22]. For example, *Teens CAN* may be incorporated into classroom instruction, but was conceived with the intention of being employed within existing afterschool and youth development programs over twelve weeks. Each of the twelve modules feature experiential learning activities intended to cultivate teamwork, which is important for afterschool educational programs [68], and build knowledge, skills, and self-efficacy associated with food literacy. With *Teens CAN* primarily intended for low-income adolescents, facilitating the curriculum within afterschool programs is particularly important for introducing youth to science-based programming applicable to daily living that they otherwise would not be permitted to access [69,70,71].

Another recommendation for food literacy programming is to include peer-modeling [22]. One reason for developing *Teens CAN* was to create a curriculum to be applied in training teen teachers. A study implementing the Shaping Healthy Choices Program curricula within 4-H found that teen teachers were inadequate at facilitating the curricula with satisfactory program fidelity [24]. It was postulated that teen teachers required additional training, especially in regard to curricula content, before they could be competent facilitators [24]. Following participation in *Teen CAN* lessons, it is anticipated that adolescents will have improvements in relevant knowledge and skills that will enable them to effectively facilitate food literacy programming with younger youth.

Adolescents acting as teachers for younger youth, known as cross-age teaching, is a common practice within 4-H. Cross-age teaching can be beneficial for adolescents as it reinforces learning concepts for themselves in addition to building confidence in teaching [72]. In contrast to tutoring, cross-age teaching involves specific training for the teen teachers who then facilitate lessons from a given curriculum over time to a group of younger youth [73]. In particular, cooking education has been successful in a cross-age teaching model [60,74]. Cross-age teaching perpetuates observational learning and can thus improve self-efficacy for various skills, including those valuable for food preparation [34,60,74]. Additionally, these programs allow opportunities for team building and improve peer relationships [62] while also encouraging implementation of cooking skills for younger youth at home [74]. A long-term nutrition and gardening program utilizing teen teachers for elementary-aged youth provides an excellent example and highlights the scalability of a program of this nature [75,76]. Applying this model employs adolescents that are culturally competent being from the same community and living within the same contexts as the younger youth they are teaching [76].

Adolescents have been found to be as effective, if not more effective than adult educators [77]. Cross-age teaching programs provide opportunities for community service for teen teachers in addition to opportunities for improving the health and self-efficacy of participants, whether teaching or learning, and increasing opportunities for introducing food literacy concepts to youth [72,75,76,78,79]. Beyond food literacy-related constructs, cross-age teaching is also beneficial for developing leadership, critical thinking, and problem-solving skills that are essential as adolescents mature [80,81]. Educational opportunities to advance food literacy in adolescents may encourage healthy eating and have long-term implications for preventing obesity. These programs may be further strengthened by utilization of cross-age teaching to improve knowledge retention while also granting soft skill development in addition to improving the health of all involved.

A major limitation of this project was the inability to assess the impact of participating in *Teens CAN* lessons on dietary behavior of adolescents. A pilot to assess *Teens CAN* implementation employing a two-tiered cross-age teaching model was started just before the COVID-19 pandemic. This model included undergraduate students trained in learner-centered pedagogy to facilitate lessons with high school-aged adolescents in afterschool programs and subsequently, adolescents were to be mentored and trained by the undergraduate students to teach local elementary school-aged youth using garden-enhanced nutrition curricula. As this study was halted early on, it will be resumed when safe and allowable. This study will include multiple data collection timepoints to assess whether adolescents have improvements in food literacy relevant constructs, such as nutrition knowledge and cooking skills self-efficacy, after participation in *Teens CAN* lessons and whether outcomes are further enhanced after acting as teachers for youth. Additionally, anthropometrics and dietary intake data will be collected from both adolescents and younger youth throughout the school year. These data will provide valuable input on the effectiveness of *Teens CAN* individually and when integrated into a yearlong mentoring program.

## Figures and Tables

**Table 1:** Food literacy attributes [19] and components [18] by topic area in *Teens CAN*.

Lesson Topic	Attributes	Components
Agriculture	“Food and other systems” “Food attitude” “Food knowledge” “Food self-efficacy” “Infrastructure and population-level determinants” “Socio-cultural influences and eating practices”	“Access food through multiple sources and know the advantages and disadvantages of these” “Determine what is in a food product, where it came from, how to store it and use it”
Nutrition	“Dietary behavior” “Food attitude” “Nutrition knowledge” “Nutrition language” “Nutrition literacy” “Nutrition self-efficacy” “Socio-cultural influences and eating practices”	“Demonstrate self-awareness of the need to personally balance food intake” “Determine what is in a food product, where it came from, how to store it and use it” “Judge the quality of food” “Understand food has an impact on personal wellbeing”
Cooking	“Cooking self-efficacy” “Food attitude” “Food knowledge” “Food language” “Food techniques” “Food self-efficacy” “Food skills across the lifespan” “Socio-cultural influence and eating practices”	“Apply basic principles of safe food hygiene and handling” “Determine what is in a food product, where it came from, how to store it and use it” “Make a good tasting meal from whatever food is available” “Make feasible food decisions which balance food needs with available resources” “Join in and eat in a social way” “Plan food intake so that food can be regularly accessed through some source, irrespective of changes in circumstance or environment” “Prioritize money and time for food”

**Table 2:** Learning concepts and objectives for *Teens CAN*.

Lesson	Learning Concepts	Learning Objective (Youth Will Be Able to…)	Evidence of Learning (Youth Were Able to…)
Agriculture 1	Food supply chain	Organize components of the food supply chain	Complete a complex flowchart detailing steps of the food supply chain
Agriculture 2	Agricultural systems	Compare and contrast various agricultural systems	Present details of four different agricultural systems and discuss their similarities and differences
Agriculture 3	Agroecology Technology	Assemble a timeline of the various movements and advancements that have shaped the food system today	Organize various food system innovations in chronological order and utilize them as inspiration to describe potential solutions for agricultural hazards
Agriculture 4	Food availability Food access	Investigate deficits that exist in some food systems and ways to improve those deficits	Build and renovate mock neighborhoods to improve the food environment
Nutrition 1	MyPlate Food groups	Evaluate why consuming a variety of foods is needed to help an individual meet their daily food group recommendations	Assess typical eating patterns of four fictional adolescents for whether food group recommendations were met and make suggestions for better adherence to recommendations
Nutrition 2	Macronutrients Micronutrients	Investigate which foods are good sources of different macronutrients and micronutrients utilizing the information provided on a Nutrition Facts Label	Analyze various foods, each containing a Nutrition Facts Label, for nutrients either meeting or exceeding 10% daily value
Nutrition 3	Nutrients of concern for underconsumption	Evaluate why consuming a variety of foods is needed to meet recommendations for nutrients of concern	Create realistic meal plans for fictional adolescents, each with a particular dietary restriction, to include recommended nutrients of concern for underconsumption
Nutrition 4	Nutrition in media	Analyze nutrition claims in the media and critique misleading information	Discuss marketing strategies being utilized in a health advertisement and use those strategies to create a factual nutrition advertisement
Cooking 1	Food safety	Identify and avoid potential food safety hazards	Correct improper food handling practices through a charades-type game and demonstrate proper food safety techniques
Cooking 2	Cooking equipment Cooking techniques	Demonstrate proper utilization of various cooking techniques and equipment	Prepare a multi-step recipe using appropriate cooking supplies and methods
Cooking 3	Seasonality Budgeting	Plan meal options that incorporate budgetary needs and seasonal produce	Draft a grocery list, adhere to a budget, and complete food shopping for four people in a mock grocery store
Cooking 4	Recipe scaling Serving and portion sizing	Plan a meal and calculate the cost per serving of that meal	Make an individualized meal and complete a guided worksheet to calculate its cost
